# Feasibility and reference intervals assessed by conventional and speckle‐tracking echocardiography in normal hamsters

**DOI:** 10.14814/phy2.14776

**Published:** 2021-03-02

**Authors:** Antonio C. L. Barros Filho, Henrique T. Moreira, Beatriz P. Dias, Fernando F. F. Ribeiro, Denise M. Tanaka, André Schmidt, Benedito C. Maciel, Marcus V. Simões, José A. Marin‐Neto, Minna M. D. Romano

**Affiliations:** ^1^ Cardiology Center of the Medical School of Ribeirao Preto University of Sao Paulo São Paulo Brazil

**Keywords:** echocardiography, hamster, left ventricular function, reference values, speckle tracking

## Abstract

**Objectives:**

This study aimed to determine feasibility, reference intervals, and reproducibility of left ventricular ejection fraction (LVEF) and speckle‐tracking echocardiography (STE) in adult Syrian hamsters.

**Background:**

Syrian hamster is an experimental model for several heart diseases. Echocardiography allows the evaluation of structure and function with bidimensional conventional techniques and STE. However, there is no data regarding reference values for bidimensional LVEF and myocardial strain in hamsters.

**Methods:**

A total of 135 female Syrian hamsters were anesthetized and studied with a small animal dedicated echocardiography system. Echocardiography measurements were obtained from M‐mode and B‐mode images. Feasibility and 95% reference intervals were obtained for LVEF using three different approaches: LVEF_Teichholz (from M‐mode linear measurements), LVEF_BMode (from area‐length method), and LVEF_ STE (from strain), and for global longitudinal (GLS), circumferential (GCS), and radial (GRS) endocardial strain. Reproducibility was assessed as intra‐class correlation coefficients.

**Results:**

Feasibility of LVEF and endocardial strain was high (95% in FEVE_Teichholz, 93% in the LVEF_BMode, 84% in the LVEF_STE, 84% from PSLAX, and 80% from PSSAX). Values of LVEF_Teichholz were significantly higher than values of LVEF_BMode, and LVEF_STE‐derived methods (59.0 ± 5.8, 53.8 ± 4.7, 46.3 ± 5.7, *p* < 0.0001). The 95% reference intervals for GLS, GCS, and GRS were respectively −13.6(−7.5;‐20.4)%, −20.5 ± 3.1%, and + 34,7 ± 7.0%. Intra‐class correlation coefficients were 0.49 – 0.91 for LVEF measurements, 0.73 – 0.92 for STE, with better results for LVEF_Teichholz and GLS.

**Conclusions:**

Evaluation of LVEF by several methods and STE parameters is feasible in hamsters. Reference intervals for LVEF and STE obtained for this experimental animal model can be applied at future research.

## Graphical abstract

Speckle tracking echocardiography (STE) is highly feasible in Syrian hamsters; Reference intervals of parameters of systolic function in hamsters are described; Reproducibility of STE in hamsters could guide future studies in this animal model.

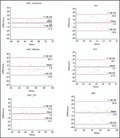

## 1 INTRODUCTION

Syrian hamsters have been used as an experimental model to study the physiopathology and effects of therapy in several cardiac diseases, some of them are not well reproduced in other rodents (Bilate et al., 2003; Crespo et al., 2011; Oliveira et al., 2016). Among non‐invasive methods for in vivo analysis of cardiac structure and function, echocardiography is unique due to its combination of high accuracy, practicality, and availability, associated with the low operating cost (Egemnazarov et al., 2015; Romano et al., 2012; Rottman et al., 2007; Scherrer‐Crosbie & Thibault, 2008).

Systolic function can be analyzed by calculating left ventricular ejection fraction (LVEF) and fractional shortening (FS), parameters already well defined in various experimental animal models (Pimentel et al., 2012). Also, myocardial deformation can be measured with speckle‐tracking echocardiography (STE), a technique enabling the detection of incipient myocardial changes (Barbosa et al., 2014; Bhan et al., 2014; Haberka et al., 2015; Saccheri et al., 2013; Zoroufian et al., 2014) which has been increasingly incorporated into clinical practice (Lang et al., 2015; Mor‐Avi et al., 2011; Voigt et al., 2015).

However, there are no data regarding reference values for myocardial strain in Syrian hamsters. The aim of this study was to determine feasibility, reference intervals, and variability of parameters derived from conventional and STE regarding LV structure and function in healthy Syrian hamsters.

## 2 METHODS

### 2.1 Experimental animals

A sample size of at least 120 animals was established based on suitable confidence intervals for non‐parametric variables (Friedrichs et al., 2012; Horowitz, 2008).

Twelve‐week‐old female hamsters (*Mesocricetus auratus*) were assessed by echocardiography. Exclusion criteria were inability to induce sedation with a recommended dose of anesthetics, clinical evidence of extra‐cardiac pathologies, presence of any cardiac arrhythmias such as ventricular and supraventricular tachycardia, and evidence of cardiac abnormalities such as congenital defects or regional hypokinesis. The research protocol was approved by the institution's Animal Research Ethics Committee (nº 025/2016).

### 2.2 Echocardiography

A commercially available and dedicated high‐resolution ultrasound system for experimental animal studies was used to perform echocardiography examinations, Vevo® 2100 (VisualSonics Inc), with a 30‐MHz linear transducer. All images were acquired by single examiner accredited for human clinical echocardiography and also with experience in performing small animal echocardiography.

After weight quantification and sedation as previously described, (Tanaka et al., 2016) chest trichotomy was performed. Animals were positioned on a tilting platform (VisualSonics Inc) with temperature control to avoid hypothermia. Electrocardiographic (ECG) signals were obtained during cardiac imaging.

### 2.3 Conventional echocardiography

Video cine loops were acquired from at least three cardiac cycles in the following projections: (Bilate et al., 2003) parasternal long‐axis (PSLAX) and (Oliveira et al., 2016) parasternal short‐axis (PSSAX) at the papillary muscle level. Static images were acquired in M‐mode, guided by the two‐dimensional images in PSLAX view, obtained perpendicularly to the LV wall, according to previous recommendations for assessing cardiovascular function in small animals models (Ram et al., 2011).

Measurements were performed at the Vevo® Lab workstation (VisualSonics Inc). Representative images of the cardiac cycle without interference of respiratory movements were obtained by M‐mode technique, allowing the measurement of interventricular septum, LVEDD, LVESD, and left ventricle posterior wall dimensions. Left ventricle mass (LV mass) was estimated from Devereux formula (Devereux et al., 1986). LVEDD, LVESD, and LV mass were presented also as indexed measures when divided by animal weight. LV fractional shortening (FS) was estimated subtracting LVESD from LVEDD and divided by LVEDD and presented as percentage. LVEF_Teichholz was estimated using linear dimensions and Teichholz method (Meller et al., 1979). To calculate the LVEF_BMode, a representative video of the PSLAX view with the best definition of the endocardium walls was selected. The frame with the largest diastolic volume was selected, and endocardial border was traced at diastole. At the same cardiac cycle, the frame with the lowest systolic volume was chosen, and the endocardial border was traced at end‐systole. Area measurements from tracings were converted to volumes using the Area‐length method (Ram et al., 2011). Bidimensional ejection fraction (LVEF_BMode) was then estimated subtracting LVESV from LVEDV and divided by LVEDV and presented as percentage.

### 2.4 Speckle‐tracking echocardiography (STE)

**FIGURE 1:**
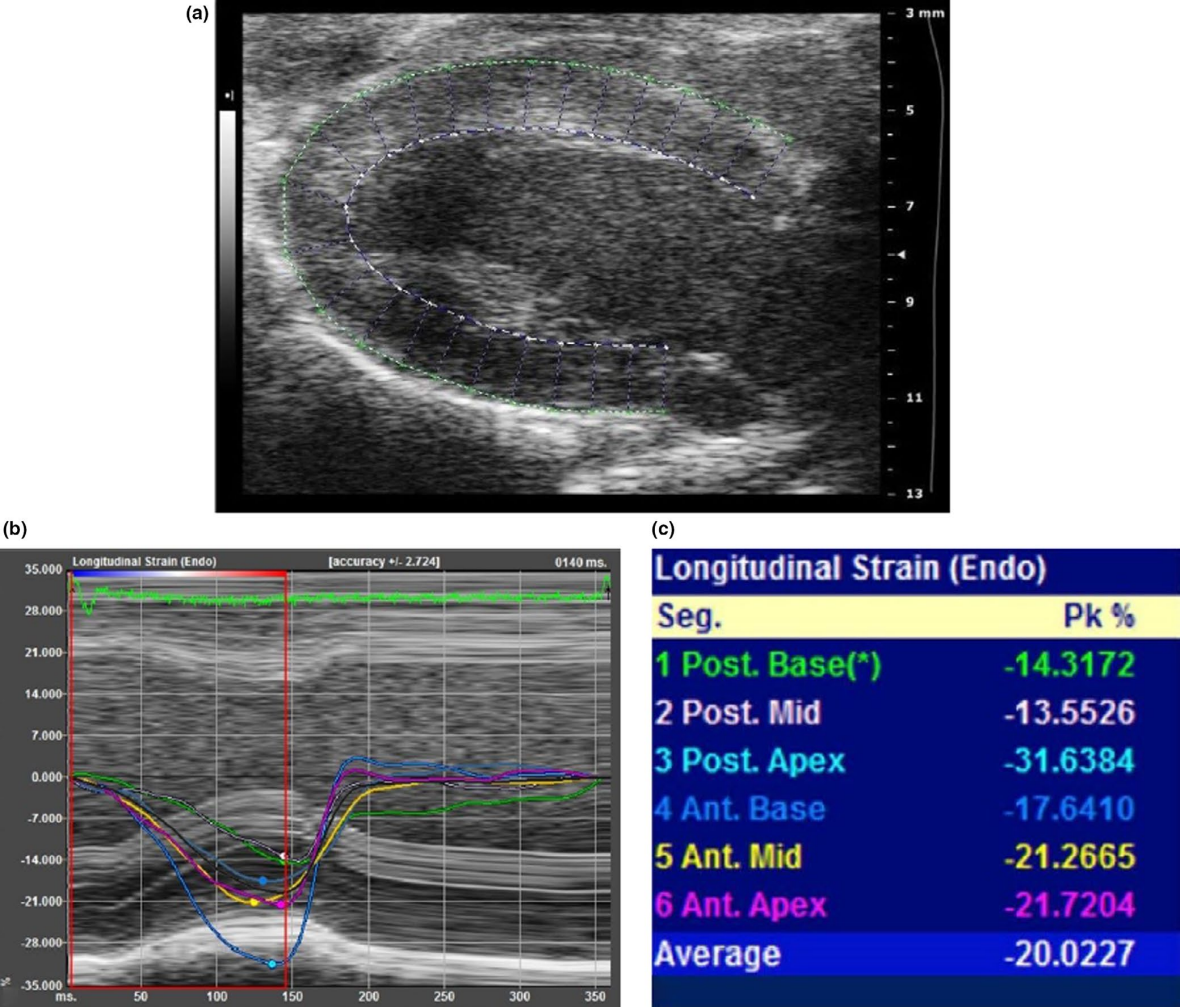
Myocardial strain analysis in hamsters from parasternal long‐axis view (PSLAX). (a) Analysis of endocardial (white dashed line) and epicardial (green outline) tracings; (b) longitudinal strain curves; (c) longitudinal peak systolic strain values and segmental nomenclature. Endocardial strain values are represented for global (white line = average of all segments), posterior basal (1); posterior mid (2); posterior apex (3); anterior basal (4); anterior mid (5); and anterior apex (6) segments

**FIGURE 2:**
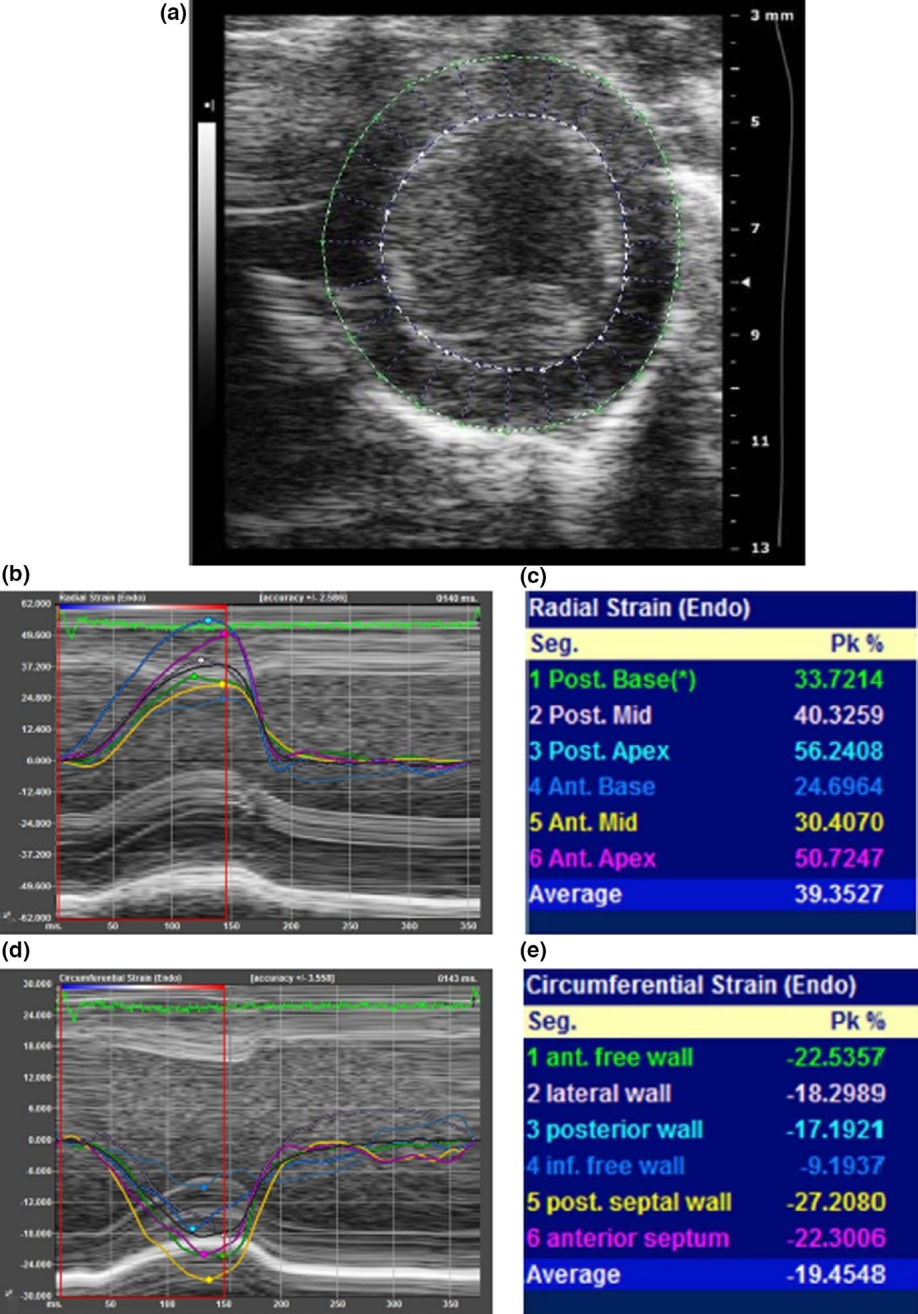
Myocardial strain analysis in hamsters from parasternal short‐axis view (PSSAX). (a) Analysis of endocardial (white dashed line) and epicardial (green outline) tracings; (b) radial strain curves; (c) radial strain peak systolic values; (d) circumferential strain curves; (e) circumferential strain peak systolic values and segmental nomenclature. Endocardial strain values are represented for global (white line = average of segments), anterior (1); lateral (2); posterior (3); inferior free (4); posterior septal (5); and anterior septal (6) segments

Bidimensional images from PSLAX and PSSAX views were analyzed using Vevo® Strain workstation (VisualSonics Inc), following the manufacturer's recommendations and based on a protocol previously described in mice (Bauer et al., 2011). PSLAX offered the global (GLS) and regional values of longitudinal strain from following segmentation: anterior basal, anterior mid, anterior apical, posterior apical, posterior mid, and posterior basal. PSSAX allowed the global radial (GRS) and circumferential strain (GCS) and regional strain from the following segmentation: anterior, lateral, inferior free, posterior, posterior septal, and anterior septal walls (Figures 1 and 2). Software offered STE analysis from both endocardium and epicardium layers. The best representative cardiac cycle image of each animal was selected for this analysis (Morgan et al., 2004). Initial systolic time was defined at the peak R wave of the ECG data. End‐systolic time was defined as the point of smallest dimension of the LV cavity. The edges of the endocardium and epicardium were manually traced sequentially using 8–12 points along the left ventricular cavity by the examiner. Tracing at diastole and systole allowed calculation of LVEF from STE technique (LVEF_STE), after the software transformed areas into volumes similarly to what is made to calculate LVEF_BMode. Myocardial strain analysis was performed in a specific panel, and tracking was provided by the software and visually checked by the examiner from both PSLAX and PSSAX views (Supplemental material). Peak systolic strain values for each segment were obtained in each projection. Blind analyses were performed independently by two trained observers to test inter‐observer variability and at two different time points by the same observer to test intra‐observer variability.

### 2.5 Feasibility analysis

Images were considered feasible for conventional measurements when they presented good quality and allowed the correct identification of the structures to be measured. For STE, feasibility was considered when LV segments were adequately tracked along the cardiac cycle by STE. Global strain from each view was considered feasible when at least five out of six segments were properly tracked, and its value was established as the mean deformation of the feasible segments, according to the ASE/EACVI recommendations (Voigt et al., 2015).

### 2.6 Statistical analysis

Reference intervals (RI) were obtained following recommendations from American Society of Veterinary Clinical Pathology (Friedrichs et al., 2012). Normal distribution of variables was evaluated by histograms and tested with Shapiro–Wilk method. Continuous variables were expressed as mean ± SD when normal distributed or by median and interquartile range (IQR) if non‐normal distribution. LVEF from different methods were compared with Student's *t*‐test or Mann–Whitney *U*‐test as appropriate. Correlation between LVEF methods was tested using Pearson test: 95% RI were determined by non‐parametric analysis when *n* ≥ 120 or by Horn method (Horn et al., 1998) when 80 ≤ *n* < 120. Linear regression analysis was used to test the influence of HR and animal weight on echocardiographic conventional and STE parameters. Significance was set as *p* < 0.05. All analyses were done using STATA Software v14 (Stata Corp, USA) and MedCalc v15 (MedCalc Software).

## 3 RESULTS

A total of 135 female Syrian hamsters were selected, and 126 were evaluated with echocardiography. Causes of exclusion were death during anesthesia (Crespo et al., 2011), thoracic deformity (Bilate et al., 2003), and evidence of echocardiographic abnormalities such as myocardial segmental hypokinesia (Crespo et al., 2011) or arrhythmias (Oliveira et al., 2016). Sample characteristics such as age, weight, heart rate, and frame rate of acquisition from PSLAX and PSSAX images are described in Table 1.

**TABLE 1:** Sample characteristics

	(*n* = 126)		
	Mean or median SD or IQR	Minimum	Maximum
Age (days)	89 (87–90)	84	96
Weight (g)	137.4 ± 12.8	112	167
PSLAX view			
Heart rate (bpm)	204.0 ± 23.0	156	256
Frame rate (fps)	186 (168–196)	158	259
PLSAX view			
Heart rate (bpm)	197.5 ± 142	142	259
Frame rate (fps)	213 (182–259)	158	289

Abbreviations: bpm, beats per minute; fps, frames per second; g, grams; IQR, interquartile range; SD, standard deviation.

### 3.1 Feasibility evaluation

**FIGURE 3:**
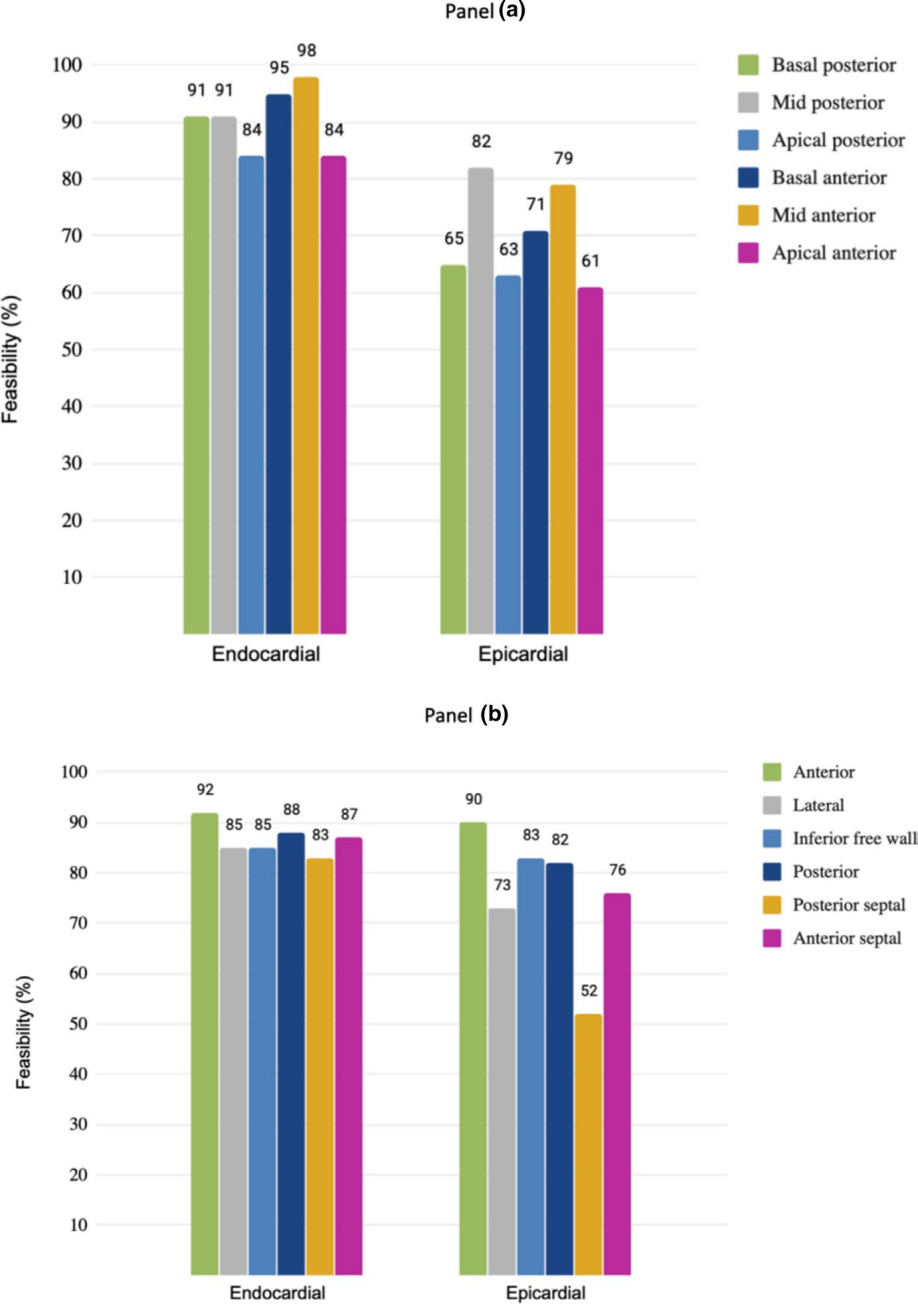
Panel (a) feasibility of PSLAX strain per layer and segment. Panel (b) feasibility of PSSAX strain per layer and segments. Feasibility is expressed by percentage

LVEF measurement was 95% feasible by MMode technique (LVEF_Teichholz and FS), 93% feasible by bidimensional echocardiography (LVEF_BMode), and 84% by STE (FEVE_STE). Feasibility was described for both epicardial and endocardial global strain parameters (GLS, GRS, and GCS). Results per segment are shown in Figure 3. Endocardial strain, measured as global indices of GLS (PSLAX) or GRS/GCS (PSSAX), showed good feasibility (84% and 80% respectively). However, epicardial strain global indices GLS (PSLAX) or GRS/GCS (PSSAX) presented low feasibility of 42% and 62% respectively. Regional strain values, both from PSLAX (GLS) and PSSAX (GRS/GCS), were also more feasible when using the endocardial layer than the epicardial layer. Endocardial regional strain from apical segments presented the lower indices of feasibility (84%) from PSLAX, whereas regional strain values from posterior‐septal segment were only 83% feasible from PSSAX (Figure 3).

### 3.2 Reference intervals

Reference intervals for conventional echocardiographic and endocardial STE parameters are described in Table 2.

**TABLE 2:** Reference intervals for conventional echocardiographic and endocardial STE parameters

	Mean/SD or median/IQR	RI 95%
LVEDD (mm)	6.5 (6.2–6.8)	[5.7; 7.4]
LVEDD/weight (mm/Hg)	4.7 ± 0.4	[3.6; 5.9]
LVESD (mm)	4.4 ± 0.4	[3.6; 5.2]
LVESD/weight (mm/Hg)	3.2 ± 0.3	[2.2; 4.2]
LV mass (mg)	263 ± 46	[171; 357]
LV mass/weight (mm/Hg)	194 ± 32	[109; 279]
Fractional shortening (FS)	32.7 ± 4.1	[21.5; 43.5]
LVEF_Teichholz (%)	59.0 ± 5.8	[47.4; 70.8]
LVEF_BMode (%)	53.8 ± 4.7	[44.3; 63.1]
LVEF_STE (%)	46.3 ± 5.7	[34.4; 57.6]
GLS (%)	−13.6 (−7.5; −20.4)	[−7.9; −19.2]
GCS (%)	−20.5 ± 3.1	[−14.3; −26.7]
GRS (%)	34.7 ± 7.0	[+21.0; +49.4]

Abbreviations: GLS, global longitudinal strain; GCS, global circumferential strain; GRS, global radial strain; IQR, interquartile range; LV, left ventricle; LVEDD, LV end‐diastolic diameter; LVEF, LV ejection fraction; LVESD, LV end‐systolic diameter; RI, reference interval; SD, standard deviation.

**FIGURE 4:**
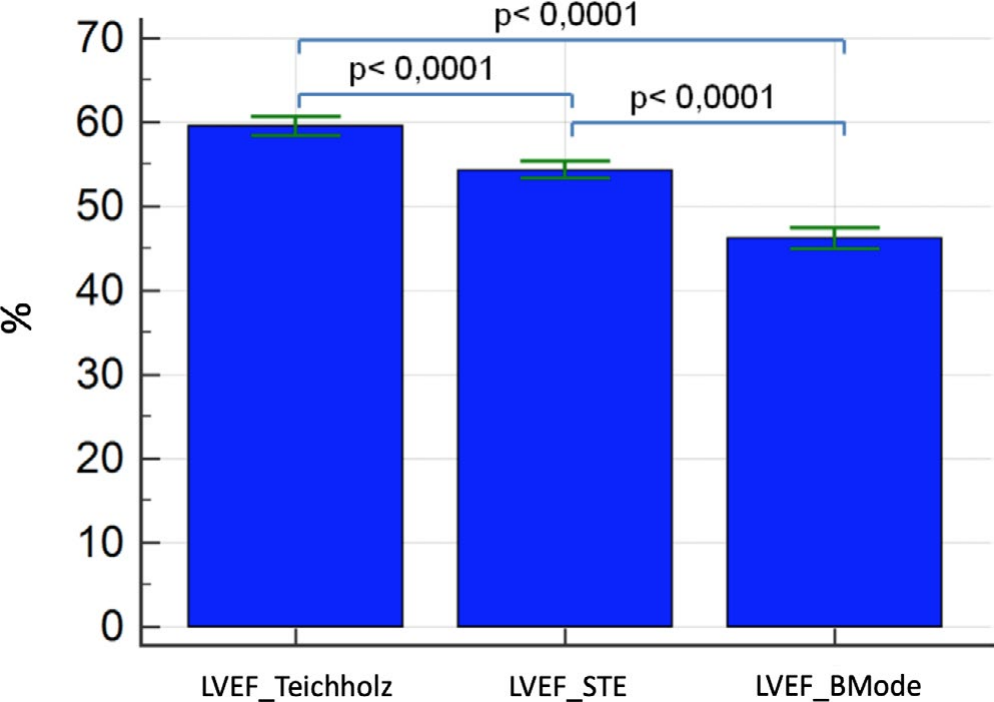
Mean values of Left ventricular ejection fraction from different methods of volume quantification: Teichholz, BMode, and STE. BMode, Left ventricle ejection fraction from bidimensional mode using area‐length method; STE, Left ventricle ejection fraction from speckle‐tracking analysis to delineate endocardial border

All conventional echocardiographic variables presented normal distribution. LVEF differed significantly between three methods (LVEF_Teichholz, LVEF_BMode, and LVEF_STE; *p* < 0.001). LVEF_Teichholz presented the higher mean values (59 ± 5.8%) compared with LVEF_BMode (53.8 ± 4.7%) and LVEF_STE (46.3 ± 5.7%) (Figure 4). A positive significant correlation between LVEF_BMode and LVEF_STE was observed (*r* = +0.43; *p* < 0.001).

Linear regression analysis showed that animal weight influenced LVEDD (*p* = 0.001), LVESD (*p* = 0.004), and LV mass (*p* < 0.001). HR influenced LVEDD (*p* = 0.0221), LVESD (*p* = 0.0003), FS (*p* = 0.0016), and LVEF obtained with all methods (*p* < 0.001). HR also influenced Endocardial STE variables, GLS, and GCS (*p* = 0.003 and *p* < 0.0001 respectively), but GRS was not. STE variables were not influenced by animal weight.

### 3.3 Variability analysis

**FIGURE 5:**
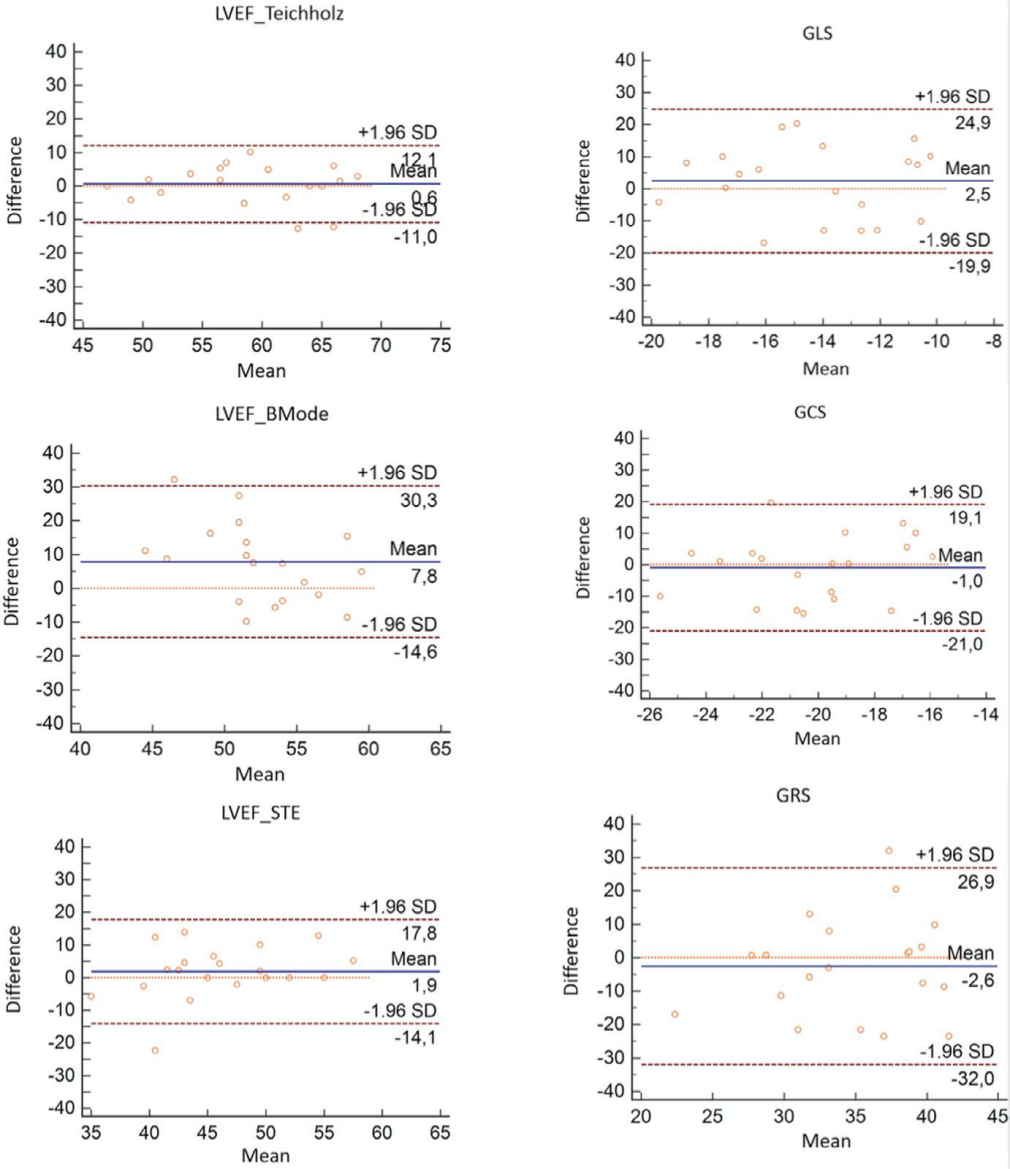
Bland Altman dispersion graphics. Intraobserver analysis for left ventricular ejection fraction measurements (LVEF_Teichholz, LVEF_BMode and LVEF_STE) and global endocardial strain variables (GLS, GCS and GRS). GCS, Global circumferential strain; GLS, Global longitudinal strain; GRS, Global radial strain; LVEF_BMode, Left ventricle ejection fraction from bidimensional mode using area‐length method; LVEF_STE, Left ventricle ejection fraction from speckle‐tracking analysis to delineate endocardial border; LVEF_Teichholz, Left ventricle ejection fraction from Teichholz formula

Intraclass correlation coefficients (ICC) were 0.91 for intraobserver comparisons of LVEF_Teichholz, 0.90 for LVEF_STE and 0.49 for LVEF_BMode. For STE variables, ICCs were 0.92 for GLS, 0.85 for GCS, and 0.73 for GRS. Interobserver ICCs was 0.86 for LVEF_Teichholz, 0.52 for LVEF_BMode, and 0.74 for LVEF_STE. For STE variables, interobserver ICC was 0.91 for GLS, 0.77 for GCS, and 0.67 for GRS. Bland‐Altman dispersion plots for LVEF measurements and STE variables are shown in Figure 5.

## 4 DISCUSSION

The present study defined feasibility and reference values of conventional and strain echocardiographic analysis of myocardial systolic function in a rodent animal model of Syrian hamsters. Measurements of global and regional endocardial LV systolic strain were also highly feasible in hamsters.

Animal experimental models have led to enhanced knowledge about several cardiac diseases by allowing the study of pathophysiological, diagnostic, and treatment aspects that are difficult to explore in clinical studies (Zaragoza et al., 2011). The Syrian hamster has been shown to be very suitable as an animal experimental model for specific cardiovascular diseases such as Chagas disease. There are, however, significant gaps in the knowledge about Chagas disease, such as who and why only some 30%–50% of chronically *T. cruzi* infected patients will develop chronic cardiomyopathy. In the Syrian hamster model, the natural history of Chagas disease can be studied through only 8 months (Oliveira et al., 2016). Conventional echocardiography measurements of LV structure and function were already studied in the hamster model of Chagas disease, but the use of clinical echocardiographic equipment and feasibility analysis have not been adequately described (Salemi et al., 2005).

STE is a novel tool to quantify myocardial deformation in any direction of LV walls. Although the STE technique is still under continuous development, (Voigt et al., 2015) it has been well studied in several cardiac conditions that evolve to myocardial dysfunction (Barbosa et al., 2014; Ersboll et al., 2014; Haberka et al., 2015; Hassanin & Alkemary, 2016; Saccheri et al., 2013; Zoroufian et al., 2014). Also, prognostic information is continually added in several clinical scenarios using STE parameters, the most robust one being the global longitudinal strain (GLS) (Sengelov et al., 2015; Takamura et al., 2011; Tang et al., 2017). However, circumferential (GCS) and radial (GRS) global parameters are still not completely studied. STE was used in other rodent models of cardiovascular diseases such as the mice model of ischemic myocardial disease (Ram et al., 2011) and was proven to be accurate in identifying myocardial damage before global reduction of LVEF (Bauer et al., 2011). Application of STE in an experimental model of Chagas disease allows the prospective study of myocardial damage through the whole evolution of disease in a highly cost‐effective time frame compared with human.

It is relevant to emphasize that most available information about STE in normal rodents was provided from control groups of studies with different scientific purposes and low number of animals (Crespo et al., ,^2008^, ^2011^, ^2012^). None of them defined the technique feasibility (Szymczyk et al., 2013), studying 10 mice, compared STE analysis with tissue Doppler analysis only in PLAX view and reported on a good correlation between both techniques. Defining feasibility of in vivo techniques such as echocardiography in small animal models is important as there are technical difficulties regarding thoracic shape, high heart rate, and animal size in each experimental model. Several studies in humans and some in other experimental animal models evaluated feasibility of STE, but none were reported in rodents (Spurney et al., 2015; Takano et al., 2015; Visser et al., 2015; Westrup & McEvoy, 2013). Takano et al. (2015) studied GCS and GRS in 33 cats, and the feasibility of such measures was 97.6%. Westrup & McEvoy (2013) in a study conducted with 54 Irish Lébrel dogs found 85.2% feasibility in regard to measurement of GLS, GRS, and GCS.

Our study aimed to analyze conventional and STE variables in a group of normal Syrian hamsters. Feasibility concept was defined based on STE clinical criteria—the presence of a non‐acceptable tracking of one out of six segments in each projection—which is a strict quality concept definition. High feasibility of global endocardial STE parameters was obtained in all projections (84% for PSLAX parameters and 80% PSSAX parameters). Regional STE parameters were less feasible at apical segments from PSLAX and at lateral, inferior free, and posterior septal segments from PSSAX projections. Recognized limitations of strain softwares to individualize‐independent speckles in lateral regions of each projection can explain these results. This is not based on a strain limitation itself, but on a limitation of echocardiographic spatial resolution on a bidimensional image. As spatial resolution is not the same in lateral portions of the beam compared with the axial portion, tracking of speckles also will not have the same accuracy and quality (Risum et al., 2012). Epicardial parameters from STE were less feasible than endocardial ones (42% for PSLAX and 62% for PSSAX). Although a gradient of strain values is known from endocardial to epicardial layers, even in human clinical studies, epicardial strain is not well consolidated (Voigt & Cvijic, 2019). Explanations about proximity with pericardium, which in fact could be a rigid structure that interferes in myocardial deformation analysis of this layer, are plausible (Unlu et al., 2018).

This paper defined reference intervals (95% RI) of normality of conventional echocardiographic measurements of ventricular geometry and function for a population of 126 normal Syrian Hamsters. Endocardial measurements of STE demonstrated feasible, hence reference intervals of normality were presented for global (GLS, GRS, GCS) parameters of STE. Consideration of statistic methods was taken to have a sample size of more than 120 animals based on suitable confidence intervals for non‐parametric variables (Friedrichs et al., 2012). Values presented here for LVDD, LVSD, LV mass, and LVFS differ from those reported in the study of (Bilate et al., 2003). Differences could be secondary to age difference of studied animals (Barbosa et al., 2014; Haberka et al., 2015), and also to a difference in anesthetic drugs used. Although isoflurane widely used can depress LVEF when evaluated by echocardiography (Pachon et al., 2015) when compared with ketamine or with ketamine/xylazine as used in our study Tanaka et al. (2016). HR of ketamine/xylazine‐anesthetized animals was lower than in Bilate study, which, in fact, may explain higher values of chamber dimensions described here.

The present study showed significant differences between values of LVEF from methods proposed (LVEF_Teichholz vs. LVEF_BMode vs. LVEF_STE). LVEF_Teichholz measurements showed the higher mean values and LVEF_STE the lowest mean values. The positive correlation between LVEF_BMode and LVEF_STE (*r* = +0.43; *p* < 0.001) probably reflects the fact that both are measured from the same bidimensional image, although with different techniques. There is no previous published data about LVEF measured by all these techniques in hamsters. It is important to consider that LVEF_Teichholz method was designed to evaluate dilated but still symmetric ventricles, and this need to be considered when applying this technique to disease models such as myocardial infarct, and especially Chagas disease (Oliveira et al., 2016). Measurements of LVEF_Teichholz and LVEF_STE presented high intraobserver and interobserver reproducibility (ICC of 0.91 and 0.86 for LVEF_Teichholz and 0.90 and 0.74 for LVEF_STE respectively). Reproducibility of LVEF_BMode showed ICC of 0.49 for intraobserver and 0.52 for interobserver analysis.

Global STE parameters as GLS and GCS showed high intraobserver and interobserver reproducibility (ICC of 0.92 and 0.91 for GLS and 0.85 and 0.77 for GCS respectively). Reproducibility of GRS measurements showed ICC of 0.73 and 0.67 respectively. This finding is similar to human studies of STE global parameters, with GLS being the more reproducible parameter (Risum et al., 2012; Voigt et al., 2015).

The lower reproducibility of GRS is technically understandable in the context of any human or small animal experimental imaging, (Tee et al., 2015) based on the same principle described for the feasibility analysis. Measurements are made from PSSAX where segments in lateral lobes of the beam do not have enough spatial resolution to provide individual speckles to be tracked (Aurich et al., 2016; Bachner‐Hinenzon et al., 2012; Voigt et al., 2015). Also, it is relevant to point out that a great deal of interest exists about the feasibility of regional myocardial strain assessment in diseases with so prominent regional impairment of LV systolic function such as ischemic heart disease or CCC (Bachner‐Hinenzon et al., 2012; Gomes et al., 2016; Oliveira et al., 2016). However, regional myocardial strain indices did not reach, until now, enough reproducibility in human studies, thus rendering segmental strain measurements jeopardized in clinical use (Bachner‐Hinenzon et al., 2012; Mirea et al., 2018; Voigt & Cvijic, 2019). In fact, although measured in this study (Supplemental material), RI for regional strain in hamsters should be taken cautiously.

Finally, this study demonstrated how HR and animal weight strongly influenced conventional echocardiographic measures of LV geometry and function. This reinforces the need to index these measures for animal weight when studying LV geometry. RI for LV dimensions indexed for animal weight was also presented in this study. Regarding the parameters of myocardial strain, HR influenced the results of GLS and GCS (*p* = 0.003 and *p* < 0.0001, respectively). However, weight did not influence any measurements of global STE.

### 4.1 Strengths and limitations

Strengths of this study included the stringent statistical methods to define RI in a large group of normal hamsters, and also the exploration of all STE measurements available from both endocardial and epicardial layers.

Some limitations need to be considered. Exclusion criteria were based on clinical and image factors, with no use of other laboratory data to detect subjacent cardiac disease. The methodology used to study normality could not address the cut off values to separate normal from pathologic conditions, and also does not include prognostic values analysis. Therefore, cautious interpretation of RI must be practiced, and these values may not be directly extrapolated to identify cardiac disease. Although this study has compared LVEF methods available for use in a small experimental animal model with a high‐resolution dedicated image equipment, it was not designed to answer the question of what is the best method among them to evaluate LV function.

### 4.2 Translational implication

Experimental animal models of cardiac disease allow researchers to explore several issues difficult to approach in clinical studies to fill important knowledge gaps regarding pathophysiology, as well as diagnostic and therapeutic issues. The Syrian hamster model is especially useful to reproduce CCC, a life‐long pathology in humans that is mimicked through 8 months with this model. Diagnostic non‐invasive in vivo tools to explore myocardial function, similar to conventional echocardiography and STE methods used in humans, would be quite welcome. Defining 95% RI for the conventional echocardiography and STE variables in this animal model allows future translational science to be applicable for understanding various cardiovascular diseases.

## 5 CONCLUSION

Conventional and STE measurements of left ventricle geometry and myocardial function using echocardiography are feasible in the Syrian hamster model of cardiovascular disease. Feasibility, reference intervals, and reproducibility of parameters of LV structure and function and STE in healthy Syrian hamsters are provided by this study.

## 6 PERSPECTIVES

Experimental animal models of cardiac disease allow researchers to explore several issues difficult to approach in clinical studies to fill important knowledge gaps regarding pathophysiology, as well as diagnostic and therapeutic issues. The Syrian hamster model is especially useful to reproduce CCC, a life‐long pathology in humans that is mimicked through 8 months with this model. Diagnostic non‐invasive in vivo tools to explore myocardial function, similar to conventional echocardiography and STE methods used in humans, would be quite welcome. Defining 95% RI for the conventional echocardiography and STE variables in this animal model allows future translational science to be applicable for understanding various cardiovascular diseases.

## CONFLICT OF INTEREST

The authors declare no conflicts of interest.

## AUTHOR'S CONTRIBUTIONS

ACLBF, HTM, FFFR, DMT, BPD, MMDR performed conception and design of the study, or acquisition of data, or analysis and interpretation of data. AS, BCM, MVS, JAMN, MMDR performed drafting the article or revising it critically for important intellectual content. MMDR did final approval of the version to be submitted.

## Supporting information



Supplementary Material
