# Supplementary material for: Feasibility and reference intervals assessed by conventional and speckle‐tracking echocardiography in normal hamsters
Source: Physiol Rep. 2021 Mar 2;9(5):e14776. doi: 10.14814/phy2.14776 (PMC7923569; doi:10.14814/phy2.14776)

How to measure myocardial strain with Vevo2100

**Step 1:** Open the software Vevo2100® (VisualSonics Inc, Toronto, Canada) and look after images from studies you want to an
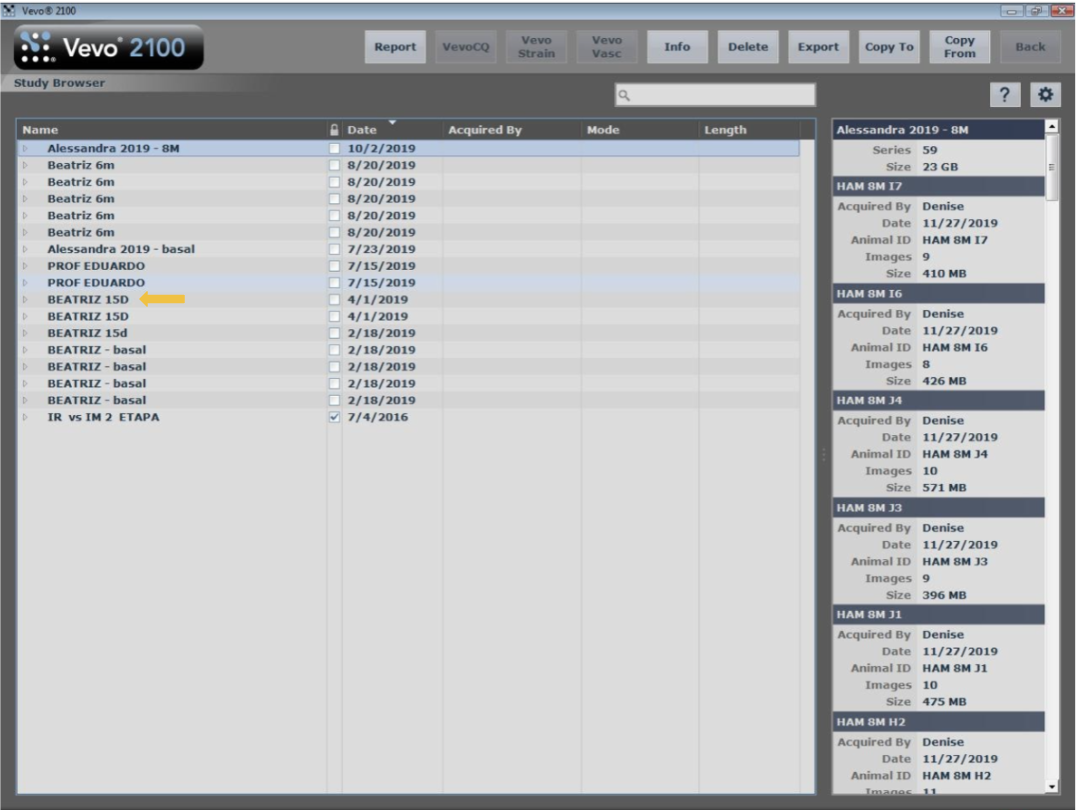

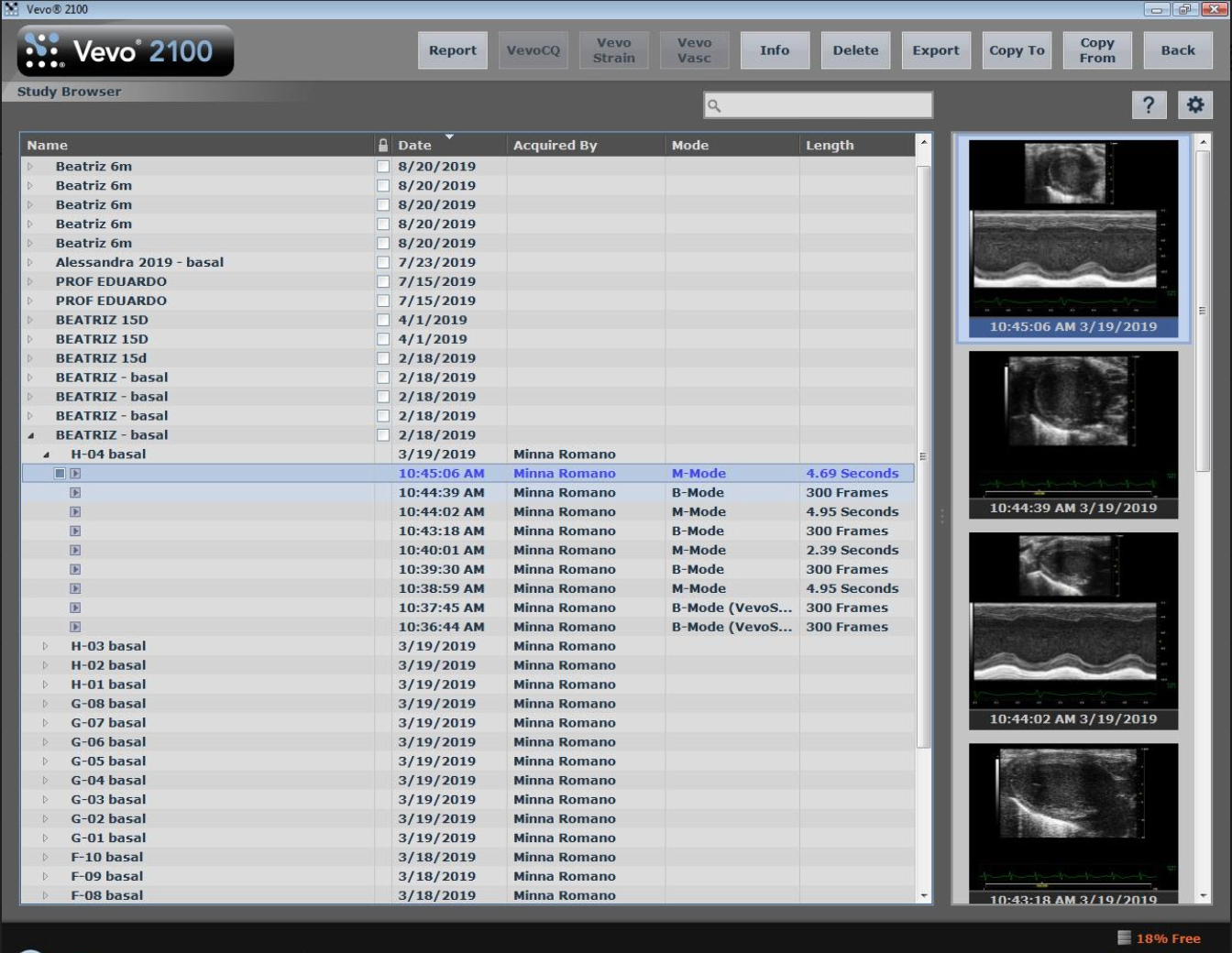
alyze (yellow arrow).

**Step 2:** Select the folder of examination and double click to open.

**Step 3:** Images from selected study will be displayed as thumbnails. Select the image of interest. Make sure images have a good ECG signal as showed here with the green line.


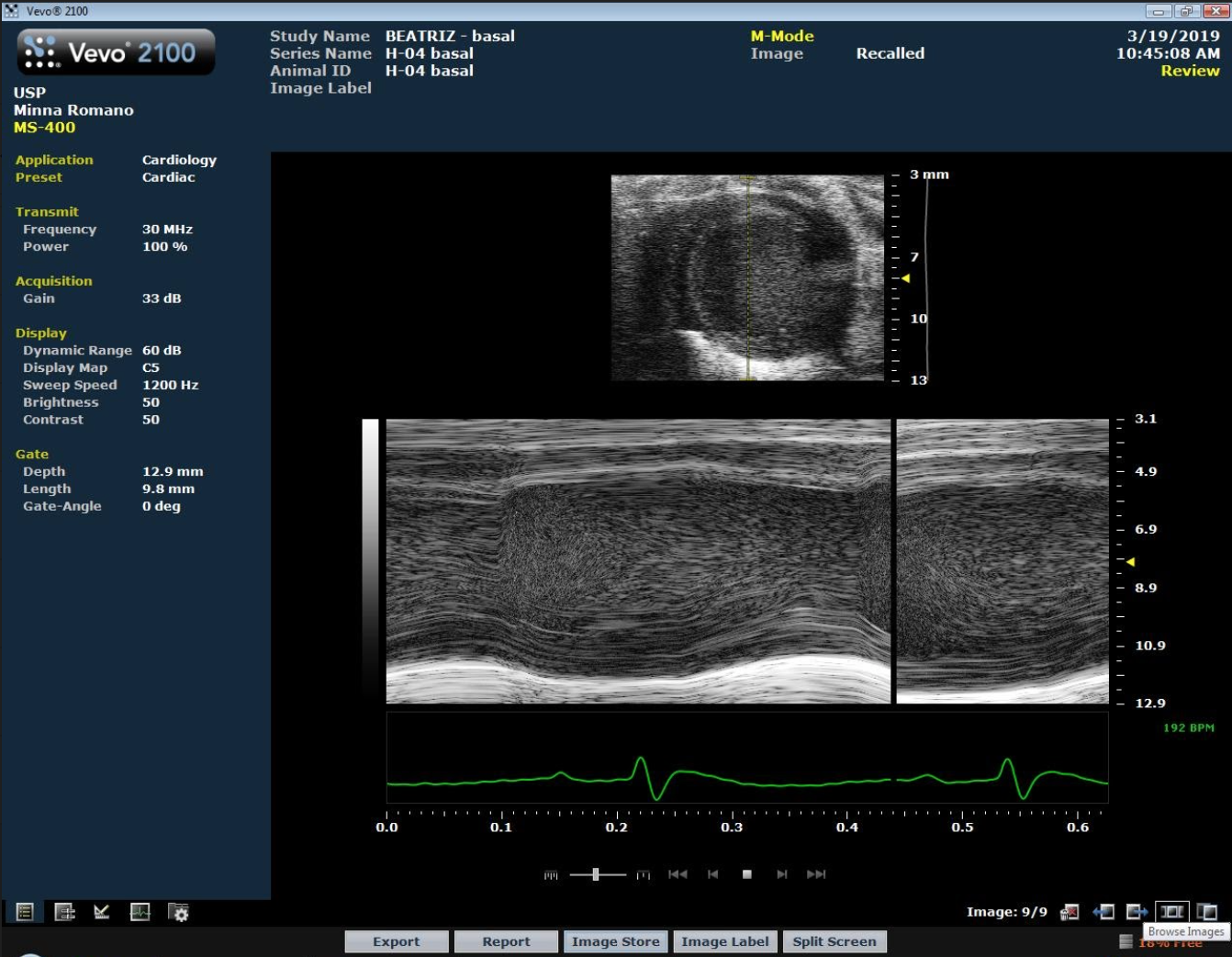


**Step 4:** After image for strain analysis is chosen, click on “Vevo Strain” button at the right superior corner of screen (yellow arrow).


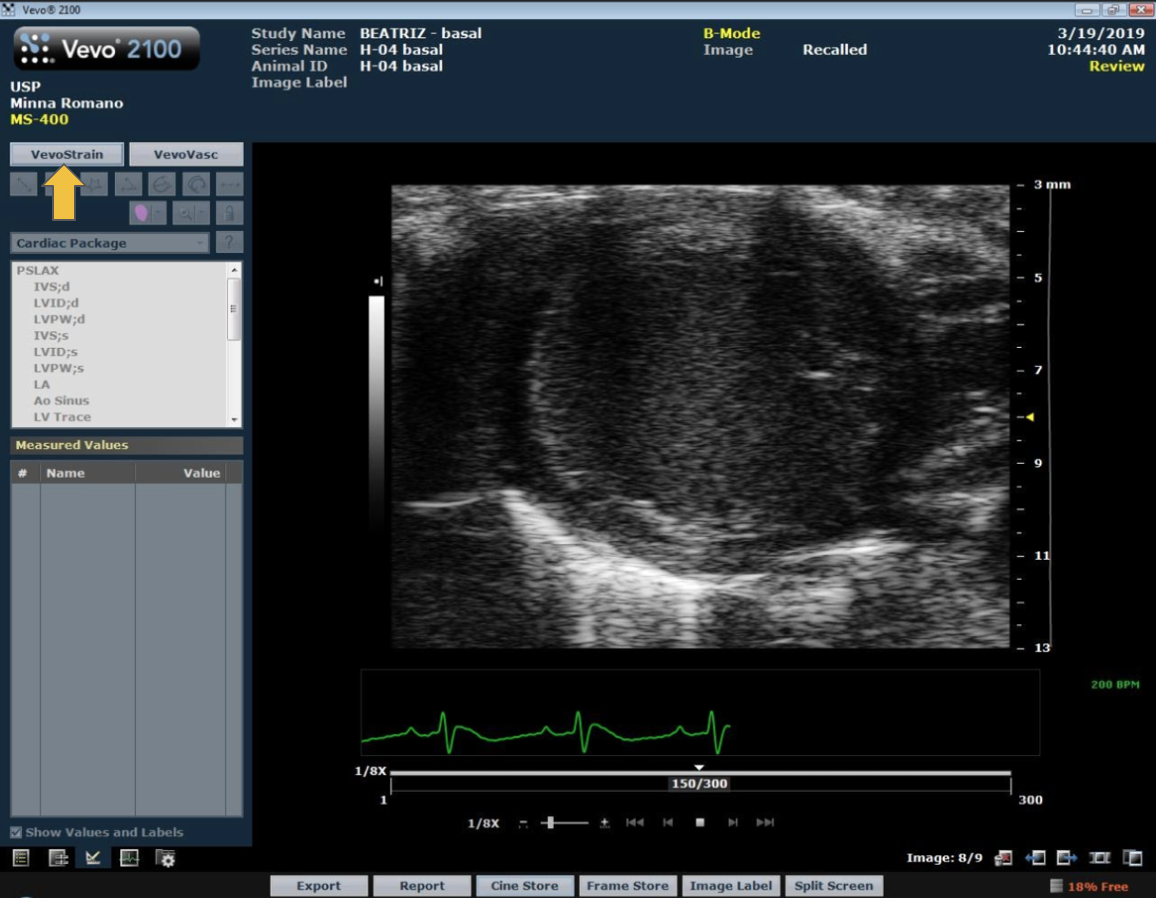


**Step 5:** Within the strain software you will need to:

1. Select one cardiac cycle of analysis. If acquisition included 3 cardiac cycles or more, to chose one cycle from the middle of capture is recommended. Avoid the first one and the last cycle.
2. Move the mouse on image and click the right button of mouse. Pull the “pen” icon so you can trace a M-Mode from the bidimensional image.
3. Click the reverse button so the background of M-Mode image will be dark making easier to identify cardiac structures as mitral valve motion or the peak systole at the more inward movement of posterior wall.
4. After this, click Next on the right superior corner of screen.


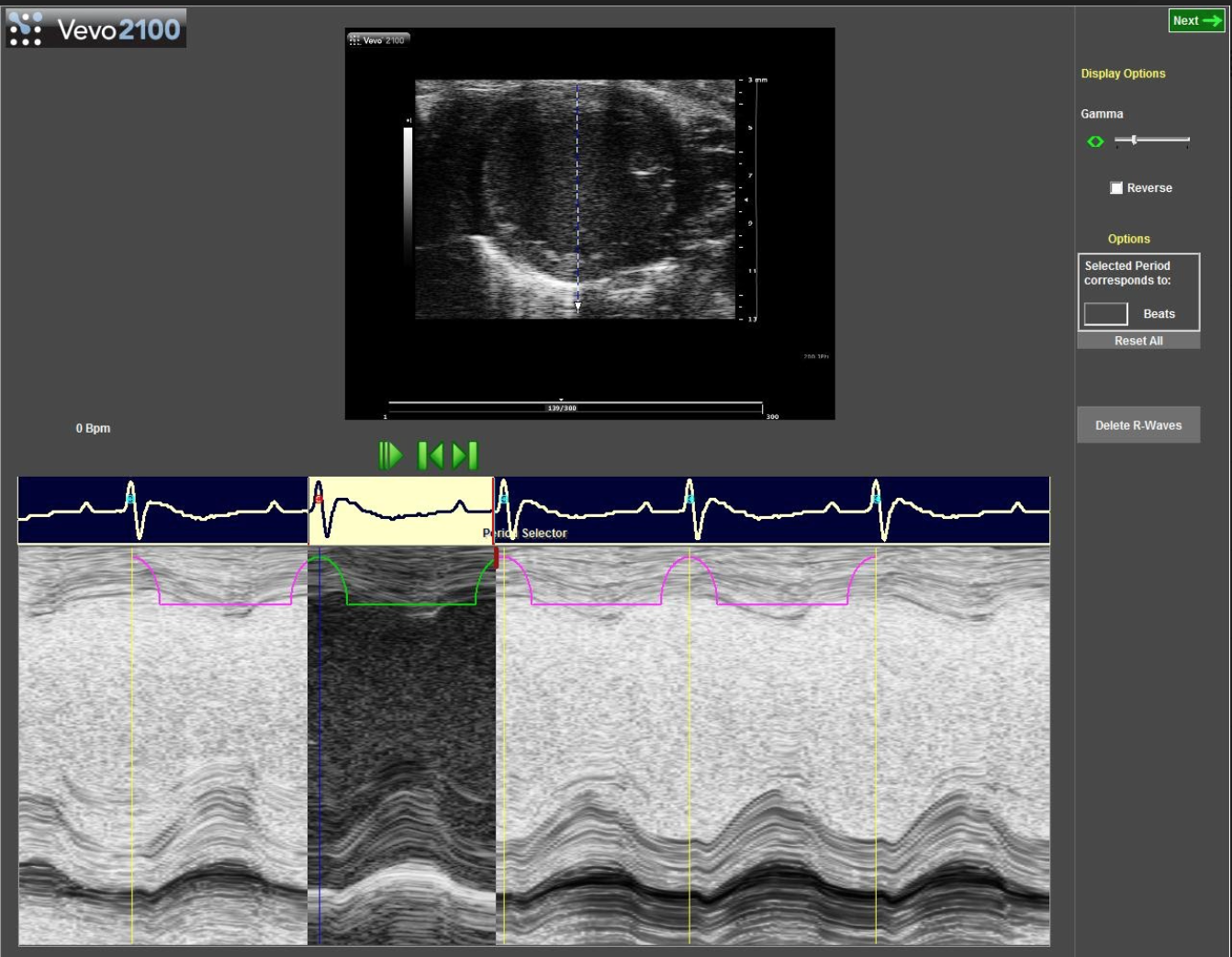


5-Now it’s time to choose what strain modality will be measured (white arrow), based on what kind of bidimensional imaging will be analyzed, if a parasternal longitudinal or short axis view. (in this case, a short axis view)


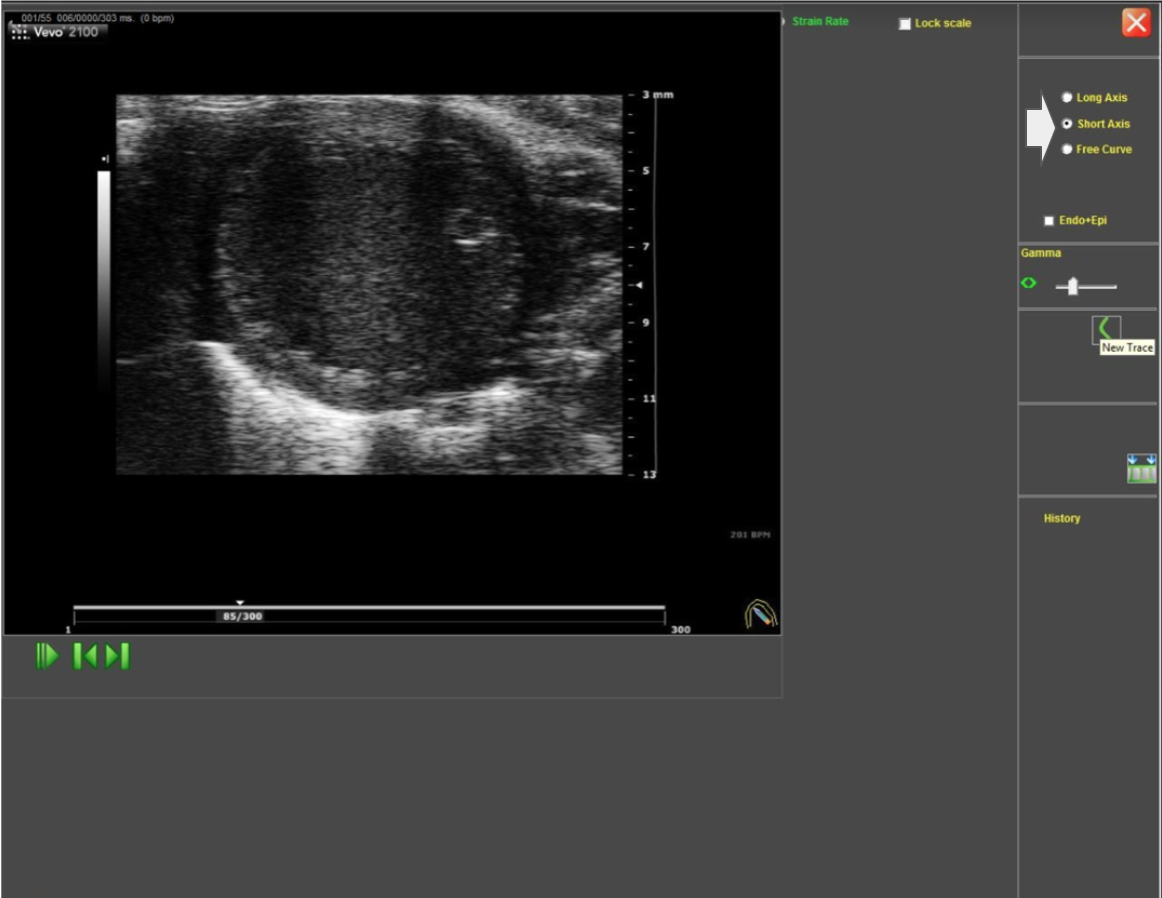


6- Click on “New trace” function (white arrow) to draw endocardial and or epicardial borders and construct the ROI (region of interest). After tracing, click on “Start Analysis” (yellow arrow). Now software will track the speckles within the myocardium to provide strain curves. This is the important time when examiner must check if the software is correctly following myocardium borders movement (tracking quality analysis). This figure represents endocardial border tracing alone, so curves will come from endocardial strain. You can choose Endo-Epi function to generate also epicardial strain if needed.
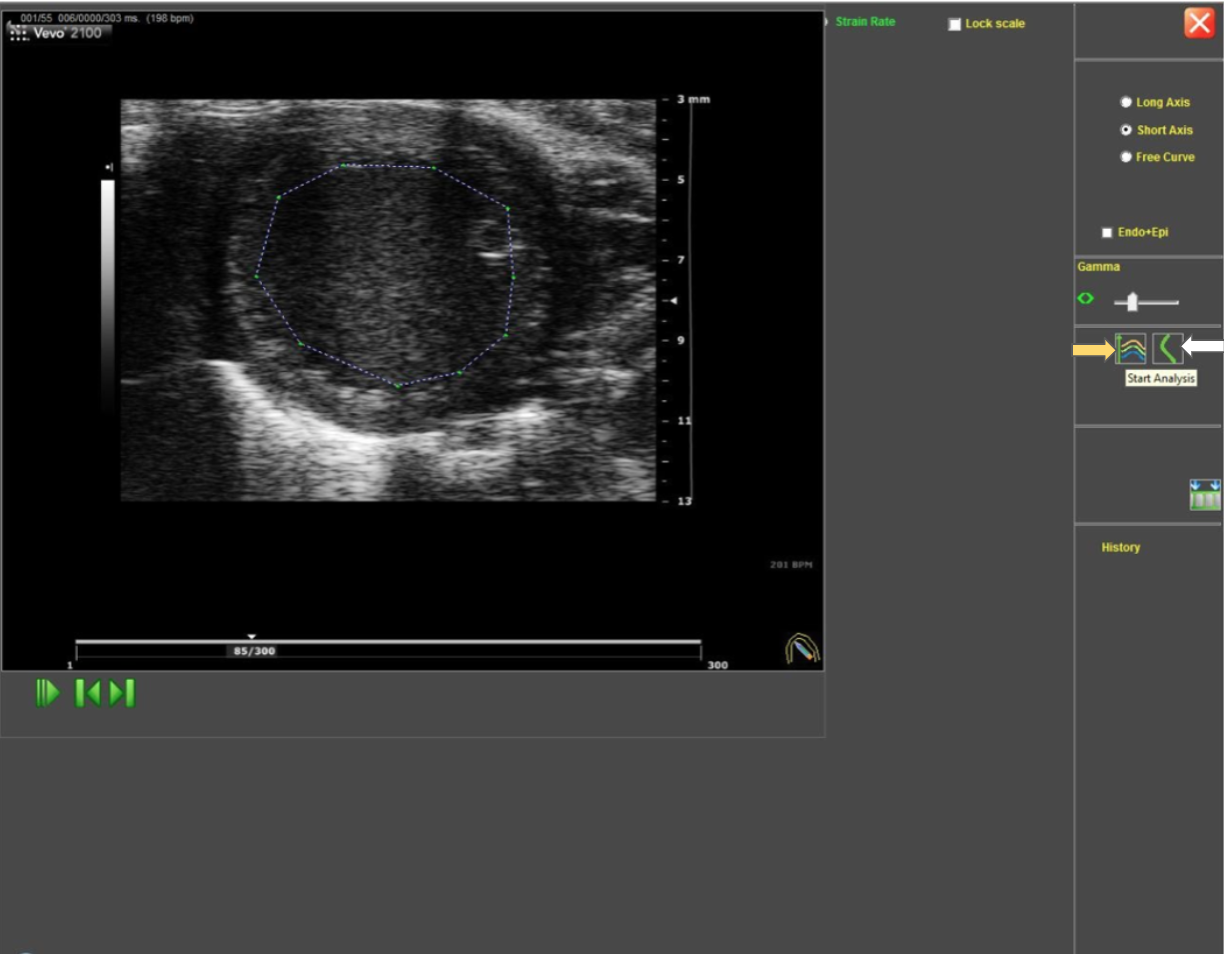


**Step 6:** Software will display the curves from each myocardial segment (white arrow), the average curve and also the bidimensional image where tracking quality should be visually checked (yellow arrow). As this is an example of parasternal short axis view, both radial and circumferential strain are analyzed. If approved analysis, click on “Time to peak analysis”.


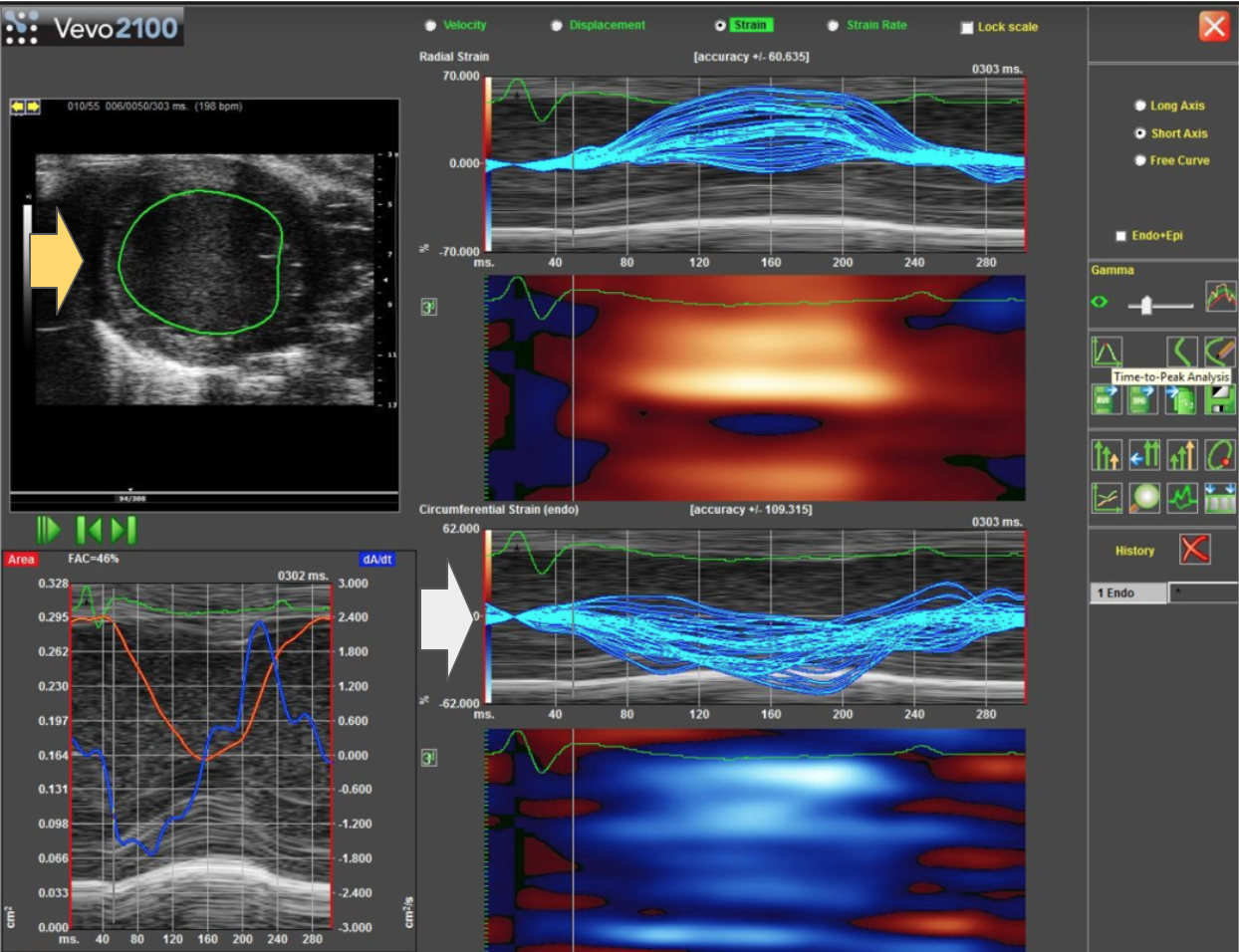


**Step 7:** Page display will include curves of strain, velocity, displacement or strain rate (green labels at the superior portion of image). Strain function is chosen in this example. Each myocardial segment curve is marked with different color. Colors match to the absolute numbers from segments displayed at the left portion of image (white arrow), Radial strain at superior portion and circumferential strain at the inferior (as this analysis is from a short axis image). If one curve does not seem adequate to you (mainly if you want to measure systolic peak and peak is not until end of systole) it can be ruled out from average calculation clicking at the right site of screen (yellow arrow).


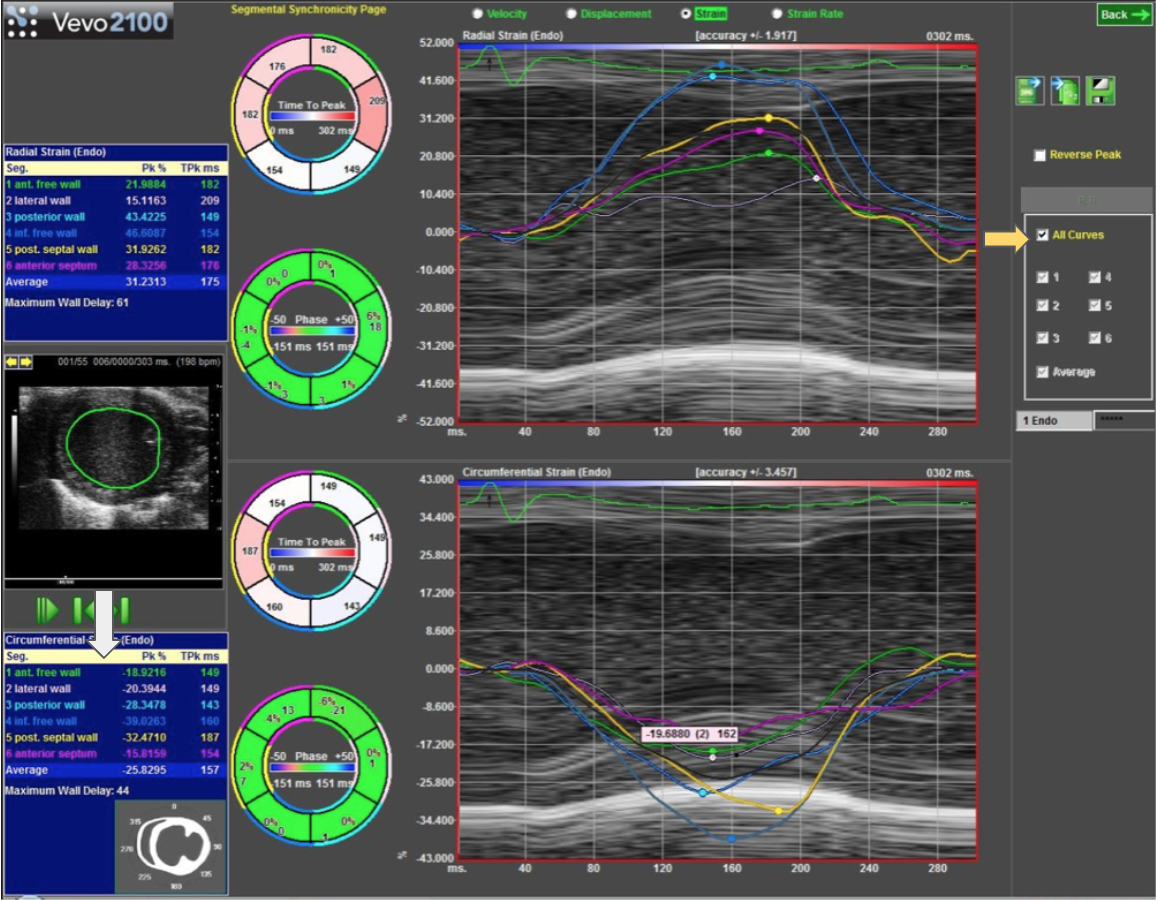


**Step 8:** After analysis concluded this can be exported to file clicking on ¨Export picture¨ on the upper right portion of screen (white arrow).
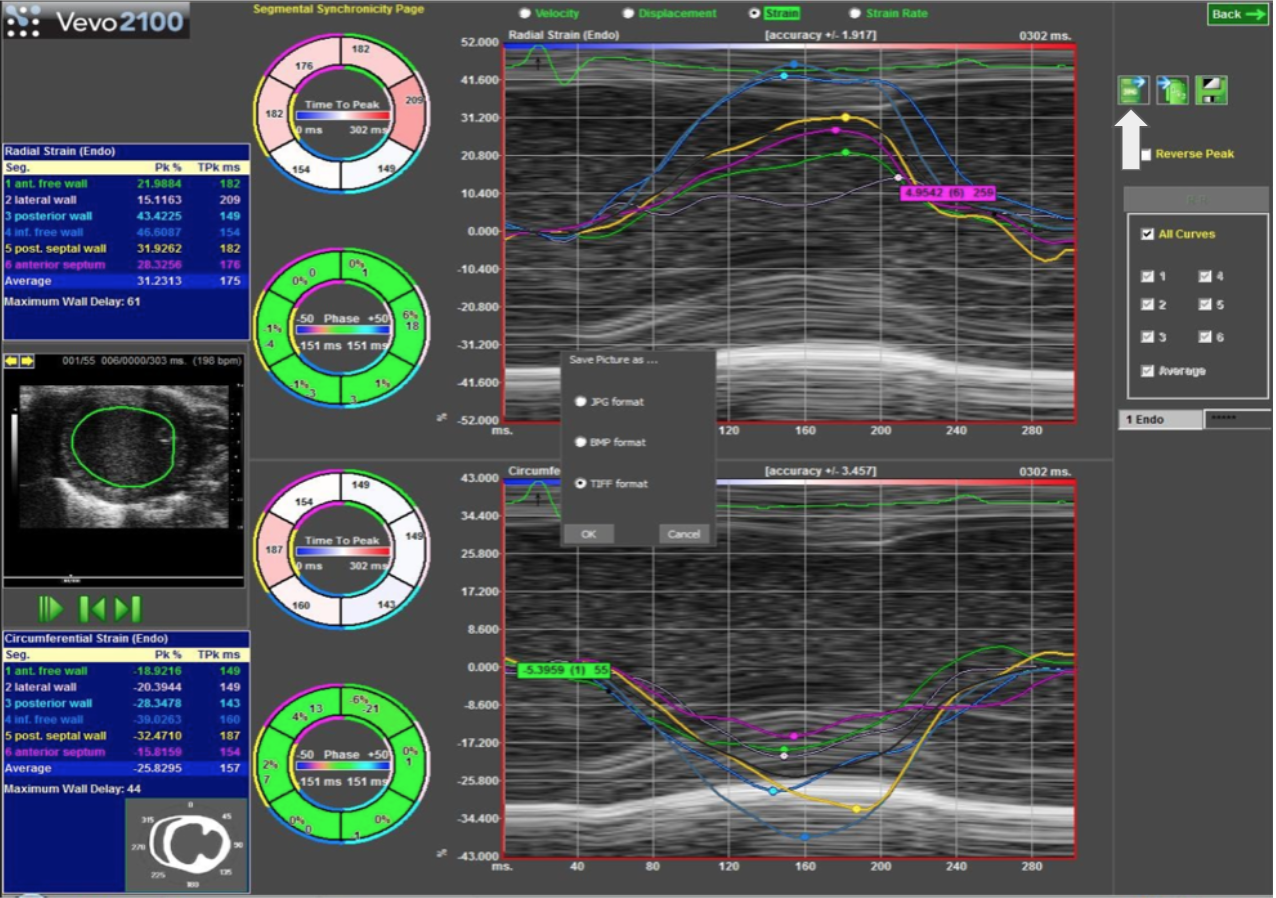

Supplement: Supplementary file 1 — Supplementary Material [file PHY2-9-e14776-s001.docx]
